# Mössbauer Spectroscopy and X-ray Diffraction Study of ^57^Fe-Labeled Tetrachloroferrate(III)-Based Magnetic Ionic Liquids

**DOI:** 10.3390/ijms12106397

**Published:** 2011-09-26

**Authors:** Rolfe H. Herber, Israel Nowik, Mirco E. Kostner, Volker Kahlenberg, Christoph Kreutz, Gerhard Laus, Herwig Schottenberger

**Affiliations:** 1 The Racah Institute of Physics, The Hebrew University of Jerusalem, Jerusalem 91904, Israel; E-Mail: nowik@vms.huji.ac.il; 2 Faculty of Chemistry and Pharmacy, University of Innsbruck, Innsbruck 6020, Austria; E-Mails: mirco.kostner@student.uibk.ac.at (M.E.K.); christoph.kreutz@uibk.ac.at (C.K.); gerhard.laus@uibk.ac.at (G.L.); herwig.schottenberger@uibk.ac.at (H.S.); 3 Institute of Mineralogy and Petrography, University of Innsbruck, Innsbruck 6020, Austria; E-Mail: volker.kahlenberg@uibk.ac.at

**Keywords:** Mössbauer, ionic liquids, metals, magnetism, crystal structure

## Abstract

Four ^57^Fe-labeled tetrachloroferrates(III) of organic cations (1-butyl-3-methylimidazolium, 1-allyl-3-methylimidazolium, 1-methyl-1-propylpyrrolidinium, tetraphenylphosphonium) were examined by temperature-dependent Mössbauer spectroscopy. The hyperfine and dynamic parameters of the iron(III) site were determined. Single crystal X-ray diffraction data of [Ph_4_P][FeCl_4_] were collected at four temperatures (295, 223, 173, and 123 K), and the dynamics of the iron atom inferred from the Mössbauer data and the single crystal *U*_i,j_ parameters have been compared.

## 1. Introduction

Magnetic ionic liquids (MILs) are low vapor pressure homogeneous phases (liquids or low-melting solids) which show a noticeable attraction to strong magnets, and have attracted considerable attention in the recent literature [1–5]. Typically, they consist of an organic cation and a tetrahalogenoferrate(III) anion [6], but other transition metal-based anions have also been reported [7,8]. A detailed Raman spectroscopic and calculational study of FeCl_3_/1-butyl-3-methylimidazolium chloride mixtures has shown that the predominant metal species is the FeCl_4_ ^−^ anion [9], a conclusion supported by visible absorption spectroscopy [10,11]. Resonant gamma ray fluorescence (Mössbauer effect, ME) and AC susceptibility data of 4-piperidinylpyridinium FeCl_4_ ^−^ were examined at low temperatures and confirmed a Néel temperature of ~2.5 K in this system [12]. The ME spectra showed a sharp transition from a rapidly relaxing paramagnetic resonance line to a six-line hyperfine spectrum between 2.50 and 2.25 K. Below 2 K, the spectra exhibited magnetic saturation. Tetraethylammonium tetrachloro-ferrate(III) was investigated by temperature-dependent Mössbauer spectroscopy and became antiferromagnetic with a Néel temperature of 3.0 K [13,14]. A survey of 164 crystal structures included in the November 2003 Cambridge Structural Database (CSD) revealed that the tetrachloroferrate ions have distorted tetrahedral structure with averaged Fe-Cl bond lengths and Cl-Fe-Cl bond angles of 2.204 Å and 109.32°, respectively [15]. It is somewhat surprising to note that there is very little reference in the ME literature to alkali metal tetrachloroferrates, and in particular to the temperature dependencies of the hyperfine and lattice dynamical parameters which have not been reported in detail.

## 2. Results and Discussion

**Figure 1:**
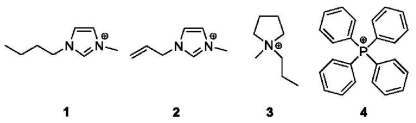
Organic cations of ^57^FeCl_4_ salts **1**–**4**.

The magnetic liquids investigated in this study showed no observable evidence of particulate matter, and no fines could be separated by centrifugation. We have examined the hyperfine parameters and metal atom dynamics of four magnetic liquids and report the related parameters of these materials. The cations associated with the tetrachloroferrate anion are listed graphically in Figure 1. The small scale synthesis of ^57^Fe-labeled compounds **1**–**4** is also described.

**Figure 2:**
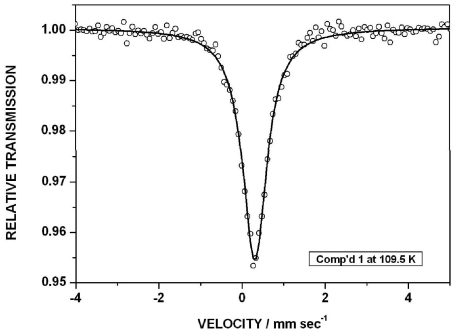
^57^Fe Mössbauer spectrum of **1** at 109.5 K. The velocity scale is with respect to the centroid of a room temperature spectrum of α-Fe. The data have been fitted to a doublet with a line width of 0.62 mm s^−1^ and a quadrupole splitting of 0.25 mm s^−1^ at 109 K.

The ME spectra in the temperature interval 5 K < *T* < 320 K consist of an asymmetrically broadened absorption line, characteristic of a relaxation-broadened transition. A representative spectrum of compound **1** is shown in Figure 2.

The hyperfine parameters at 90 K (isomer shift, *IS*, and quadrupole splitting, *QS*) as well as the derived dynamical parameters [16] (effective vibrating mass, *M*_eff_, and Mössbauer lattice temperature, *θ*_M_) are summarized in Table 1.

The line width at half maximum is generally temperature-dependent, and in several cases can be understood in terms of a fast relaxation process. In none of these spectra was it possible to extract magnetic ordering information, and it is presumed that magnetically ordered particles, when present, are sufficiently small so that the 5 K minimum temperature used in this study exceeds the blocking temperature of these particles. However, attraction of the liquid samples to a strong magnet was clearly demonstrable even above room temperature. Even molten salts **3** and **4** also exhibited attraction to a strong magnet.

### 2.1. (1-Butyl-3-Methylimidazolium) (^57^FeCl_4_) (**1**)

The hyperfine parameters (isomer shift (*IS)* and quadrupole splitting (*QS*) at 90 K are included in Table 1. The line widths of the ME spectra fitted with a relaxation spectrum program are essentially temperature-independent with an average value of 0.63 ± 0.03 mm s^−1^ suggestive of a relaxation process which is fast compared to the characteristic ME time scale. The *IS* decreases with increasing temperature, but is not well fit by a linear correlation leading to an approximate *M*_eff_ of 102 ± 13 Da and a ME lattice temperature of ~67 ± 4 K. The temperature dependence of the logarithm of the recoil-free fraction, −d(ln*f*)/dT, is [16.8(4) × 10^−3^ K^−1^] with a correlation coefficient of 0.95 for 9 data points.

### 2.2. (1-Allyl-3-Methylimidazolium) (^57^FeCl_4_) (**2**)

As above, the ME spectra consist of a single broad resonance absorption line. The *IS* and *QS* parameters are included in Table 1. The temperature dependencies of both the *IS* and recoil-free fraction are well fitted by a linear regression, leading to a value of *M*_eff_ = 87 ± 7 Da and a *θ*_M_ = 90 ± 4 K. The line width is effectively temperature independent arising from the same fast relaxation as above.

### 2.3. (1-Methyl-1-Propylpyrrolidinium) (^57^FeCl_4_) (**3**)

The ME spectra of this sample consisted not only of the broad central line associated with a paramagnetic compound, but in addition, to two minor Fe resonances presumably due to traces of impurities. These two impurities accounted for 15 and 5% of the total area under the resonance curve at 94.5 K. The influence of these impurities was corrected for in the subsequent data analysis. As in the case of the preceding compounds, the line widths of the major (paramagnetic) resonances are essentially temperature independent, again suggesting that the relaxation rate even at 92 K is fast compared to the ME time scale.

### 2.4. (Tetraphenylphosphonium) (^57^FeCl_4_) (**4**)

**Figure 3:**
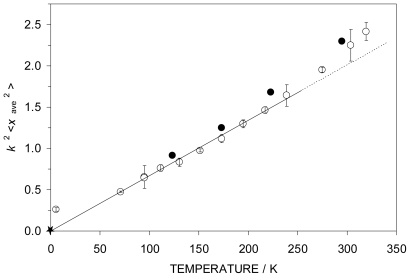
The *k*^2^<*x*_ave_ ^2^> parameter for **4** extracted from the Mössbauer data (open data points) and the single crystal X-ray data (filled data points). The *T* = 0 K point extrapolated from the linear portion of the data is indicated by the starred point as discussed in the text.

This new compound, which is not an IL, was intended as a crystallographic reference with high symmetry and only weak interactions between the ions. It is in contrast to the preceding ones in that the paramagnetic FeCl_4_ ^−^ complex is ion-paired to a phosphonium cation rather than a quaternary nitrogen atom, but except for a somewhat larger *QS* hyperfine parameter, the ME derived values are very similar to those of the salts with nitrogen containing cations. Only for this sample, it was possible to archive the Mössbauer spectra over a significantly larger range (5 < *T* < 319 K) than for the other samples studied. This made it possible to elucidate the spin relaxation mechanism in this case in detail. In addition, single crystal X-ray data were acquired at 4 different temperatures to allow a comparison with the vibrational data derived from the Mössbauer experiments. The temperature-dependent *IS* and *QS* parameters, as well as the ln*f* values, are well fitted by linear regressions (corr. coeff. = 0.999, 0.954 and 0.992 for 14 data points, respectively). The temperature-dependence of the *IS* and ln*f* parameters lead to an effective vibrating mass of 67 ± 1 Da. As has been detailed previously [17], the ln*f* data can be expressed in terms of 
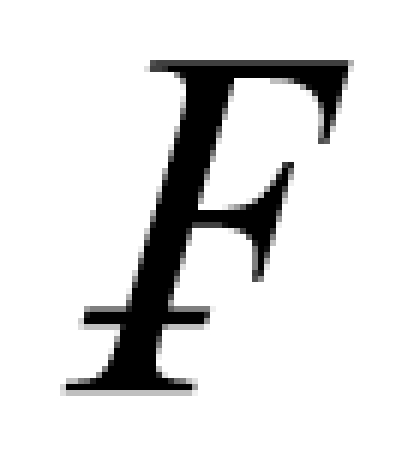
 = *k*^2^<*x*_ave_ ^2^>, where *k* is the wave vector of the gamma radiation and <*x*_ave_ ^2^> is the mean square amplitude of vibration of the metal atom. This latter quantity can similarly be calculated from the *U*_ij_ values of the X-ray diffraction data, and thus 
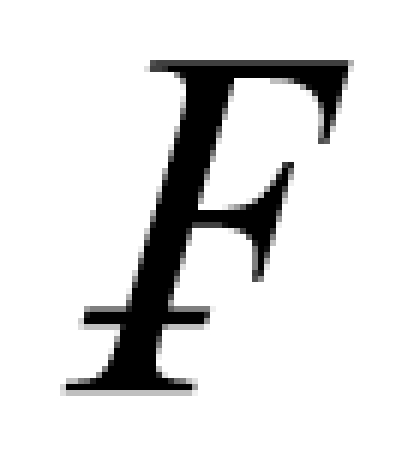
 can be compared between the two techniques. This comparison is shown in Figure 3 in which the open points are those derived from the Mössbauer experiments ( 
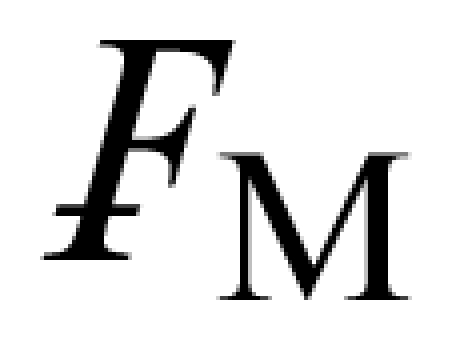
), while the filled data points refer to the X-ray data ( 
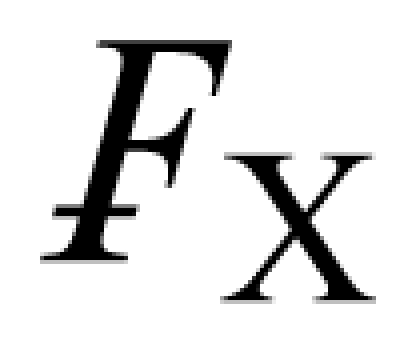
). As will be seen from the figure, the 
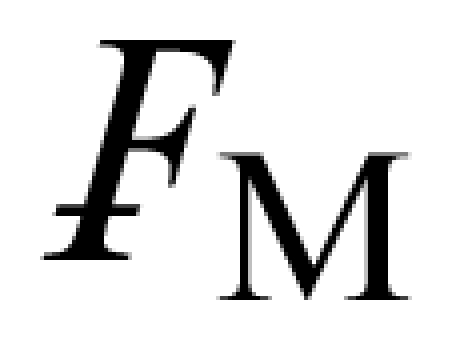
 and 
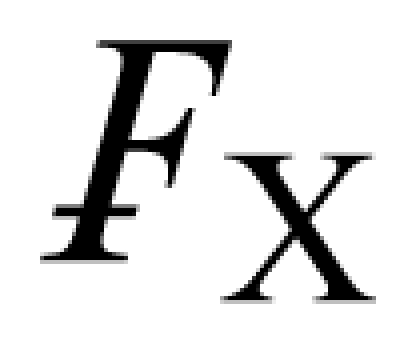
 data are in quite reasonable agreement. The departure from linearity in the high temperature regime arises from the population of low frequency vibrational (or librational) motions in the solid. The starred data point is the *k*^2^<*x*_ave_ ^2^> value when the high *T* data are extrapolated to *T* = 0 K, making the assumption that *f* → 1 in the low *T* limit.

**Figure 4:**
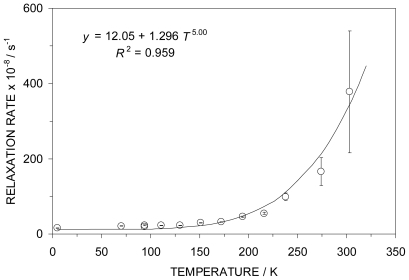
The temperature-dependence of the relaxation rate (in arbitrary units) for the paramagnetic Fe atom in **4** obeys a power law which is 5th order in *T*, indicative of a Raman spin-lattice relaxation process.

As noted above, the full width at half maximum of the ME resonance line is temperature-dependent, decreasing with increasing temperature and is indicative of a relaxation mechanism involving the metal atom spin. These temperature-dependent line widths have been analyzed using the Wickmann-Wertheim formalism [18] to yield a value of the relaxation rate, making the assumption that the effective hyperfine field is that of an *S* = 5/2 state. This relaxation behavior is summarized graphically in Figure 4 and has been analyzed in terms of a power law in *T*. The *T* dependence is of the form *T*^5.0 ± 0.7^, indicative of a Raman type spin-lattice relaxation mechanism [19].

**Figure 5:**
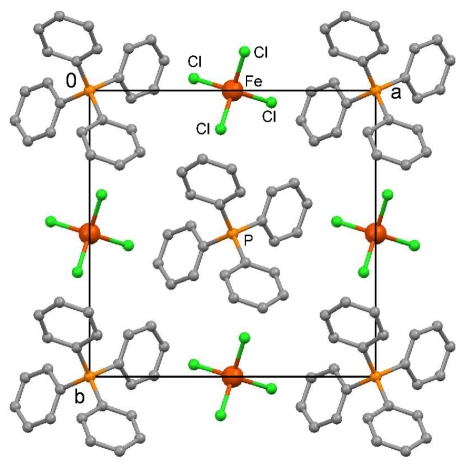
View of the crystal packing in (Ph_4_P) (FeCl_4_) in the direction of the crystallographic *c* axis at 123 K. Fe-Cl bond length 2.1939(5) Å, Cl-Fe-Cl angles 105.51(2) and 113.47(3)°. All hydrogen atoms were omitted for clarity.

As expected, X-ray crystallography of (unlabeled) **4** revealed a highly symmetrical tetragonal crystal lattice without specific interionic contacts. The ions are arranged along 4-fold rotoinversion axes in the direction of the crystallographic *c* axis. The crystallographic data are listed in Table 2. A view of the crystal packing at 123 K is depicted in Figure 5. Finally, a new survey of the CSD (June 2011) resulted in 366 FeCl_4_ ^−^ ions in 278 crystal structures, after coordinating or disordered anions were excluded, and gave an average Fe-Cl bond length of 2.184 Å and a Cl-Fe-Cl angle of 109.457° in more or less distorted tetrahedral geometries.

## 3. Experimental Section

^57^Fe powder (>95%) was purchased from Advanced Materials Technologies, Singapore. NMR spectra were recorded with a Varian Unity 500 spectrometer. Very broad signals were observed as expected from paramagnetic compounds. IR spectra were obtained with a Nicolet 5700 FT spectrometer; intensities are indicated as w = weak, m = medium, s = strong. HR MS were recorded with a Finnigan MAT 95 instrument.

### 3.1. General Procedure for the Synthesis of **1**–**4**

Hydrochloric acid (0.55 mL, 12 M) was added to ^57^Fe powder (9.97 mg, 175 μmol). Hydrogen evolution started immediately, and the mixture was stirred for 48 hours at room temperature in contact with air. Then, the corresponding chloride salt of **1**–**4** (1 equation) was added to the solution, and the product separated (**1**, **2** as liquid; **3**, **4** as solid). The mixture was stirred for another 15 minutes, and the product was extracted with dichloromethane. The solution was dried over MgSO_4_, filtered, and the solvent evaporated. Volatiles were removed in vacuum to give the hygroscopic products which were stored in Schlenk tubes under nitrogen.

### 3.2. (1-Butyl-3-Methylimidazolium) ^57^FeCl_4_ (**1**)

Yield: 43 mg (63%) as a brown liquid. ^1^H NMR (CD_2_Cl_2_, 500 MHz): *δ* 1.27, 1.84, 2.87, 3.81, 5.49, 7.47, 9.31. IR (neat): 3148 w, 3116 w, 3101 w, 2960 w, 2934 w, 2873 w, 1591 w, 1563 m, 1460 m, 1382 w, 1162 s, 1101 w, 830 m, 740 s, 648 w, 620 s cm^−1^. FAB MS [M^+^] *m/z* = 139.15 (th. 139.12). FAB MS [M^−^] *m*/*z* = 196.76, 198.76, 200.75, 202.75 (th. 196.81, 198.81, 200.81, 202.80).

### 3.3. (1-Allyl-3-Methylimidazolium) ^57^FeCl_4_ (**2**)

Yield: 46 mg (81%) of a yellow-brown oily liquid. ^1^H NMR (CD_2_Cl_2_, 500 MHz): *δ* 6.06, 6.73, 7.80, 9.07. IR (neat): *ν* = 3152 w, 3114 w, 3095 w, 1592 w, 1562 m, 1446 w, 1421 w, 1159 s, 1105 w, 990 w, 947 m, 830 m, 741 m, 674 w, 620 s cm^−1^.

### 3.4. (1-Methyl-1-Propylpyrrolidinium) ^57^FeCl_4_ (**3**)

Yield: 47 mg (82%) of a yellow solid. M.p. 97–104 °C. ^1^H NMR (CD_2_Cl_2_, 500 MHz): *δ* 2.95, 4.27. IR (neat): *ν* = 2971 m, 2939 w, 2879 w, 1458 s, 1303 w, 1261 w, 1038 w, 1002 m, 970 w, 936 m, 883 w, 817 w, 756 m cm^−1^. FAB MS [M^+^] *m/z* = 128.19 (th. 128.14).

### 3.5. (Tetraphenylphosphonium) ^57^FeCl_4_ (**4**)

Yield: 93 mg (98%) of a yellow solid. M.p. 200–208 °C. ^1^H NMR (CD_2_Cl_2_, 500 MHz): *δ* 8.02. IR (neat): *ν* = 1591 w, 1485 m, 1437 m, 1340 w, 1170 w, 1106 s, 996 m, 924 w, 742 w, 721 s, 683 s cm^−1^. FAB MS [M^+^] *m/z* = 339.11 (th. 339.13). FAB MS [M^−^] *m*/*z* = 196.77, 198.77, 200.76, 202.74 (th. 196.81, 198.81, 200.81, 202.80).

### 3.6. X-ray Crystallography

X-ray diffraction data of (unlabeled) **4** were acquired with an Oxford Diffraction Gemini-R Ultra diffractometer using *ω* scans at four temperatures (295, 223, 173, and 123 K). Intensity data were recorded using graphite-monochromated Mo*K*_α_ radiation *(λ* = 0.71073 Å) and refined on *F*^2^. Standard strategies were employed for data collection, cell refinement and data processing. Hydrogen atoms were placed in calculated positions. CCDC 792734-792737 contain the supplementary crystallographic data for this paper. These data can be obtained free of charge from The Cambridge Crystallographic Data Centre [20].

### 3.7. Mössbauer Spectroscopy

Temperature-dependent Mössbauer experiments were carried out as previously described [21], using a ^57^Co(Rh) source in transmission geometry. Spectrometer calibration was effected using a 20 mg cm^−2^ α-Fe absorber at room temperature, and all isomer shifts are referred to the centroid of such spectra. Sample preparation depended on the nature of the sample. The liquid samples (**1** and **2**), which were supplied as small droplets resulting from the enriched ^57^Fe synthesis, were taken up in a few drops of CH_2_Cl_2_, transferred to a Perspex sample holder, and the excess solvent was removed by evaporation in a vacuum dessicator. These samples, consisting of thin films, were then rapidly cooled in liquid nitrogen and transferred cold into the cryostat. Solid samples (**3** and **4**) were mixed with a small amount of BN and transferred, as is, to Perspex sample holders. The temperature of the cryostat was monitored over the data collection intervals (up to 24 hours per temperature point) using the Daswin software [22]. The transmission counting rate was monitored before and after each temperature point to assure no sample loss. For compound **4**, low temperature spectra at 5, 70, and 94 K were acquired using a closed cycle cryostat (Janis Model SHI-850-5) in transmission geometry.

## 4. Conclusions

In this study, four “magnetic ionic liquids” and related solids have been examined by temperature-dependent Mössbauer spectroscopy. In all cases, the common anionic constituent is the ^57^FeCl_4_ ^−^ tetrahedral structure, and the hyperfine and dynamical properties of this moiety have been elucidated over a significant temperature range. The spectra consist of a broad absorption which is characteristic of a paramagnetic iron site relaxing by spin-spin or spin-lattice processes. The *IS* values at 90 K fall into a narrow range (0.31–0.33 mm s^−1^) characteristic of high spin Fe(III). Similarly, the *QS* values occupy a narrow range and are comparable to values reported in the literature [12,23]. Neither of these two parameters appears particularly sensitive to the structure of the cationic part of the salts which evidently involve only weak interactions between the anion and the organic cation. The lattice dynamics of the metal center in tetraphenylphosphonium tetrachloroferrate(III) (**5**) have been determined over a wide temperature range, and the 
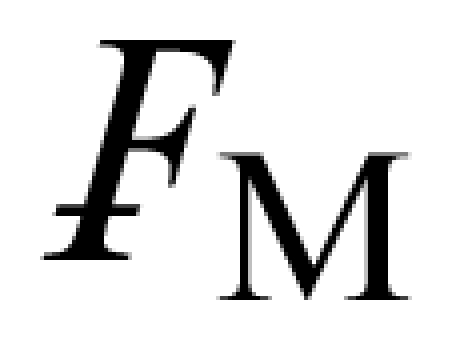
 values have been found to be in excellent agreement with the 
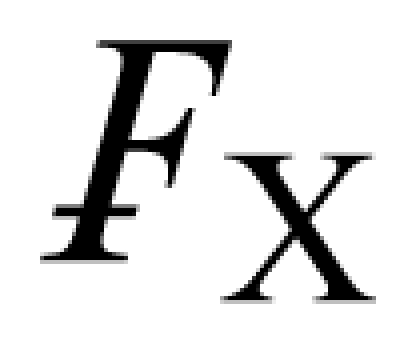
 data extracted from single crystal X-ray data. The iron atom in this compound is found to relax by a Raman process, the relaxation rate depending on *T*^~5^.

## Figures and Tables

**Table 1:** Hyperfine Parameters of **1**–**4** at 90 K.

	1	2	3	4
*IS*(90)/mm s^−1^	0.310(7)	0.310(4)	0.311(6)	0.333(8)
*QS*(90)/mm s^−1^	0.211(7)	0.187(7)	0.234(8)	0.392(8)
*M*_eff_/Da	102 ± 13	87 ± 7	79 ± 10	67 ± 1
*θ*_M_/K	67 ± 4	90 ± 4	120 ± 9	131 ± 1

**Table 2:** Crystal data and refinement details of **4** at different temperatures.

*T*/K	295	223	173	123
CCDC no.	792734	792735	792736	792737
Empirical Formula	C_24_H_20_P·Cl_4_Fe			
Formula Weight	537.02			
Crystal System	tetragonal			
Space Group	*I* 4̄			
*a*, *b*/Å	12.9906(6)	12.9701(6)	12.9515(6)	12.9483(5)
*c*/Å	7.2022(6)	7.1128(6)	7.0548(5)	7.0090(5)
*V*/Å^3^	1215.41(13)	1196.54(13)	1183.38(11)	1175.12(11)
*Z*	2			
*D*_c_/g cm^−3^	1.467	1.491	1.507	1.518
*μ*/mm^−1^	1.14	1.15	1.17	1.17
*F*(000)/e	546			
Crystal Size/mm	0.32 × 0.32 × 0.24			
*θ*_max_/°	29.2	29.3	29.4	29.2
Index Range	−16 ≤ *h* ≤ 17; −17 ≤ *k* ≤ 17; −8 ≤ *l* ≤ 9	−14 ≤ *h* ≤ 16; −17 ≤ *k* ≤ 16; −7 ≤ *l* ≤ 9	−16 ≤ *h* ≤ 14; −17 ≤ *k* ≤ 13; −9 ≤ *l* ≤ 9	−11 ≤ *h* ≤ 16; −16 ≤ *k* ≤ 17; −8 ≤ *l* ≤ 9
Reflections Collected	5151	4853	5040	4742
Independent Reflections, *R*_int_	1478, 0.031	1471, 0.030	1474, 0.033	1446, 0.027
Reflections Observed (≥ 2*σ*)	1079	1301	1300	1382
Absorption Correction	multi-scan			
*T*_min_, *T*_max_	0.893, 1	0.986, 1	0.971, 1	0.937, 1
Data/Restraints/Parameters	1478/0/69	1471/0/69	1474/0/69	1446/0/69
Goodness of fit on *F*^2^	0.89	1.07	1.00	1.03
Final *R*_1_, *wR*_2_ [(*I* > 2*σ*(*I*)]	0.029, 0.061	0.033, 0.064	0.027, 0.056	0.026, 0.050
Final *R*_1_, *wR*_2_ (all data)	0.049, 0.063	0.041, 0.070	0.034, 0.057	0.028, 0.052
Largest Diff. Peak, Hole/e Å^−3^	0.32, −0.29	0.44, −0.43	0.37, −0.40	0.26, −0.23
Flack Parameter	0.01(2)	−0.01(2)	0.026(19)	0.016(19)
